# Predicting co-morbidities in chemically sensitive individuals from exhaled breath analysis

**DOI:** 10.2478/v10102-012-0020-7

**Published:** 2012-09

**Authors:** Harold I. Zeliger, Yaqin Pan, William J. Rea

**Affiliations:** 1 Zeliger Chemical, Toxicological and Environmental Research, West Charlton, New York, USA; 2 Environmental Health Center – Dallas, Dallas, Texas, USA

**Keywords:** chemical sensitivity, sequential absorption, toxic chemicals, chemical mixtures, exhaled breath analysis

## Abstract

The exhaled breath of more than four hundred patients who presented at the Environmental Health Center – Dallas with chemical sensitivity conditions were analyzed for the relative abundance of their breath chemical composition by gas chromatography and mass spectrometry for volatile and semi-volatile organic compounds. All presenting patients had no fewer than four and as many as eight co-morbid conditions. Surprisingly, almost all the exhaled breath analyses showed the presence of a preponderance of lipophilic aliphatic and aromatic hydrocarbons. The hydrophilic compounds present were almost entirely of natural origin, i.e. expected metabolites of foods. The lipophile, primarily C3 to C16 hydrocarbons and believed to have come from inhalation of polluted air, were, in all cases, present at concentrations far below those known to be toxic to humans, but caused sensitivity and signs of chemical overload. The co-morbid health effects observed are believed to be caused by the sequential absorption of lipophilic and hydrophilic chemicals; an initial absorption and retention of lipophile followed by a subsequent absorption of hydrophilic species facilitated by the retained lipophile to produce chemical mixtures that are toxic at very low levels. It is hypothesized that co-morbid conditions in chemically sensitive individuals can be predicted from analysis of their exhaled breath.

## Introduction

Exhaled breath analysis has been shown to successfully predict the presence of certain diseases in man, including diabetes, transplant rejection, and some cancers (Pauling *et al.*, 1971; Phillips *et al.*, 2003a, b; Phillips *et al.*, 2004a, b; Corradi *et al.*, 2010). In all the cases cited for such purposes in the literature, the exhaled breaths of the patients involved contained mixtures of lipophilic and hydrophilic chemicals (Silbergeld *et al.*, 2011).

Concentrations of chemicals contained in exhaled air, alveolar air and surrounding ambient less polluted air were obtained via gas chromatography and mass spectrometry (GC/MS) for more than 400 chemically sensitive patients who presented at the Environmental Health Center – Dallas. All of these patients presented with between four and nine co-morbid conditions. It was anticipated that the chemical compositions and concentrations of these chemicals in the exhaled breath could be predictive of chemical sensitivity and other co-morbid conditions.

## Subjects and methods

Of the more than 400 patients, the records of thirty randomly chosen individuals were selected for detailed study. These individuals ranged in age from 12 to 86 years with a median age of 47.7, and 70% were female. Between 40 and 120 chemicals were identified in the exhaled breath of each patient. As the concentrations of all were low ( in the part per billion range), the top 20 in abundance were taken as significant for potential impact. The presenting patients who were analyzed for relative abundance all had between 4 and 9 distinct points of impact of the chemicals in their bodies and all had exhibited signs and symptoms of chemical sensitivity. The breath analysis samples were collected in a less polluted environmentally controlled room by the methods of Rea and Phillips (Rea 1997, Phillips 1997). Air collected from the breath sample was analyzed via GC/MS by the standard method (Phillips 1997). All patients had proven chemical sensitivity by intradermal provocative skin testing, oral or inhaled challenge (Rea 1997). Patients had no food or medication for 8 hours before the test. Their prescribed diet included less polluted food and water.

## Results

The compounds found in the exhaled breath of these patients were almost exclusively exogenous lipophilic C3 to C16 aliphatic and aromatic hydrocarbons. Hydrophilic compounds were almost all endogenous. The concentrations measured were orders of magnitude lower than the known toxic levels of these species. The abundances of the top 6 at times were relatively high, in the 200–1000 part per billion (ppb) range, but still far below the known toxicity levels for these compounds.

Since almost exclusively lipophilic exogenous compounds were identified in the analysis and because of the similar toxicological properties of these compounds, the lipophils were considered as additive and treated as such.

All presenting patients had nervous systems and immune system impacts and most had respiratory, cardiovascular and gastrointestinal impacts as well. All had a minimum of 4 and a maximum of 9 different systems affected. Table 1 shows the exhaled breath analysis data in ppb, as well as the affected systems for each of the 30 patients. Table 2 shows the number of systems impacted versus the number of individuals. All individuals demonstrated nervous system and immune system impacts. This finding is consistent with organ switch phenomenon which has been previously reported (Perbellini *et al.*, 1985; Laseter *et al.*, 1983; Rea *et al.*, 1987; Pan *et al.*, 1991).

**Table 1:** Relative abundance of breath toxins found in 30 chemically sensitive patients. Data in parts per billion (ppb).

	Less Polluted Room Ambient			
PT #	Breath	Alveolar	Air	Impacts*
1	7.6	2.6	4.8	left
2	55.0	52.5	2.1	NS IM CV CS
3	11.3	4.2	5.6	AL NS IM MK HR CS
4	563.2	499.0	64.2	NS IM CV GI CS
5	529.1	480.9	47.8	AL NS IM MK CV GI RS FR CS
6	1486.8	1420.4	54.2	AL NS IM GI RS CS
7	838.2	701.1	134.9	AL NS IM CV GI CS RS UR
8	1257.4	1176.4	78.5	AL NS IM CV GI RS CS
9	135.2	128.7	6.4	left RS
10	149.8	74.9	75.3	AL NS IM CV GI CS RS
11	615.5	539.7	73.8	AL NS IM GI RS CS
12	21.2	13.4	7.6	AL NS IM CV RS CS
13	839.6	749.9	89.6	AL NS IM MK CV GI RS CS
14	925.1	818.8	113.4	AL NS IM CV RS CS
15	156.0	151.8	6.0	AL NS IM CV CS RS
16	309.1	306.4	5.7	AL NS IM GI RS HR CS
17	447.7	427.7	25.1	AL NS IM MK RS CS
18	374.8	364.5	10.9	AL NS IM CV GI RS CS
19	35.6	33.7	1.0	AL NS IM MK CV GI CS RS
20	310.9	282.2	14.7	AL NS IM CV GI DR RS CS
21	462.7	27.2	1.8	AL NS IM CV GI RS CS
22	131.8	1.3	1.0	AL NS IM MK GI RS CS
23	528.1	478.7	17.8	AL NS IM CV GI RS CS
24	85.8	76.7	9.2	AL NS IM SK DR RS CS
25	2.6	1.1	1.5	AL NS IM CV GI RS CS
26	11.8	1.1	2.5	AL NS IM CV GI RS CS
27	11.8	7.1	4.3	AL NS IM CV GI RS CS
28	41.2	38.6	2.2	AL NS IM CV GI RS CS
29	68.3	64.2	7.5	AL NS IM GI RS CS
30	60.2	50.1	9.0	AL NS IM CV GI RS CS

AL = Allergic sensitivity; CS = Chemical sensitivity; CV = Cardiovascular effects; DR = Dermal effects; GI = Gastrointestinal effects; IM = Immune system impact effects; MK = Musculoskeletal effects; NS = Nervous system effects; RS = Upper and lower respiratory system effects

* All patients had chemical sensitivities

**Table 2:** Multiple systems involved in chemically sensitive patients.

# Systems Impacted	# of People
4	2
5	5
6	9
7	10
8	3
9	1

Table 3 lists the numbers and percentages of individuals impacted for each affected system. In addition to these effects, of the 30 individuals, 4 people (13%) had synthetic implants, 4 had viral, bacterial or fungal infections and 13 (43%) reported toxic exposures to mold, other pesticides and heavy metals.

**Table 3:** Number and percentage of individuals for each affected system.

System	# of People	%
Nervous	30	100
Immune	30	100
Chemical sensitivity	30	100
Allergic responses	28	93
Respiratory	25	83
Cardiovascular	23	77
Gastrointestinal	23	77
Musculoskeletal	10	33
EMF sensitivity	5	17
Hormonal	3	10
Dermal	2	7
Female reproductive	2	7
Urinary track	1	3
Tumor	1	3

It should be noted that lipophilic hydrocarbons are not known to attack any of the systems listed above with the exception of the nervous system, but the nervous system is not known to be impacted by these compounds at the low levels found (Jarnberg *et al.*, 1998; Kristiansen *et al.*, 1988). All these individuals could not walk a straight line or stand on their toes with their eyes open or closed, showing neurological dysfunction. All individual species were present at values far less than 1.0 part per million and some sub 1.0 part per billion.

It should also be noted that the ambient air gradient values in the controlled less-polluted test environment for many of the individual species were non-detectable. Seven patients (27%) exhaled butane and one exhaled propane, despite the fact that those species were not present in ambient air.

## Discussion

It was anticipated that certain organ/system effects could be associated with specific exhaled chemical species. Though all patients exhibited multiple system effects, this did not turn out to be the case. Essentially no exogenous hydrophilic chemicals were found in the exhaled breaths of the study group. Numerous lipophilic hydrocarbons, however, including the very low molecular weight propane and butane were exhaled by patients despite not being present in the air gradients of the room in which these individuals were studied.

It is hypothesized that the finding of lipophilic hydrocarbons in the alveolar air of individuals with zero values recorded for their ambient less polluted room air gradients is indicative of relatively long term retention of these chemicals. This hypothesis is consistent with the known toxicology of these chemicals, i.e. that they are metabolized/eliminated much more slowly than hydrophilic species (Goldstein *et al.*, 1993; Jandacek *et al.*, 2001; Monosson, 2010). Rea's work has shown that 4–7 days in a less polluted controlled environmental room are required to purge the body of some of these toxicants (Rea, 1997). It has been shown by Zeliger that lipophilic chemicals bind to lipophilic membranes in the body and facilitate the absorption of hydrophilic species that would otherwise not be absorbed (Zeliger, 2003; 2008).

It is further hypothesized that long term bonding and slow metabolism/elimination coupled with constant environmental exposure to these chemicals usually at home and or at work, establishes a steady state lipophilic coating on body membranes that can absorb hydrophilic species at any time. Accordingly, the exposure to mixtures of chemicals that are hazardous need not be simultaneous but can also occur sequentially to induce toxic effects at low concentrations (Zeliger, 2011). Therefore an individual who is constantly exposed to lipophilic chemicals has established a situation in which any subsequent exposure to toxic hydrophiles can initiate a toxic attack. This hypothesis helps explain why people often are seemingly sickened following exposures to low levels of single chemical species. It is well known that mixtures of lipophilic and hydrophilic chemicals are toxic at levels much lower than those of the individual species and that such mixtures attack organs not known to be affected by the single chemical (Zeliger 2003; 2011).

The results of this study demonstrate that chemically sensitive individuals are subject to multiple other systemic effects when their exhaled breath contains exogenous lipophilic chemicals. These results also strongly suggest that the presence of exogenous lipophilic chemicals in the exhaled breath of chemically sensitive individuals is predictive of multiple co-morbid effects, particularly of the immune and nervous systems in such individuals. Lipophilic chemicals present in the environment arise primarily from petroleum (combustion and volatilization of hydrocarbons) and tobacco smoke. The results presented here suggest that chemically sensitive individuals should avoid exposures to these sources as much as possible.

## Conclusions

Members of the subject study group were found to have numerous systemic ill health effects triggered by chemicals and having one of their diagnoses as Chemical Sensitivity. Their exhaled breath analysis revealed the presence of a preponderance of low level lipophilic hydrocarbon species, that alone are believed to be of very low toxicity, and essentially no exogenous hydrophilic species. The ill health effects induced are believed to be caused by the establishment of a steady state lipophilic layer on body membranes and subsequent exposure to toxic hydrophilic species results in the lipophile facilitated absorption of hydrophilic species that would otherwise not be absorbed. This sequential absorption of chemical mixtures is believed to be responsible for the onset of environmentally induced systemic effects in people. The presence of exogenous lipophilic chemicals in the exhaled breath of chemically sensitive individuals is believed to be predictive of neurological, immunological, respiratory, cardiovascular, gastrointestinal and other health effects in such people.

These findings point out the need to limit environmental exposures to lipophilic chemicals even at levels far below their current permissible exposure levels (PELs). It is also recommended that PELs for chemical toxicants be reduced by at least one order of magnitude and that air and water pollution allowable concentrations likewise be significantly lowered.
